# Unveiling Disparities: Analyzing Hispanic Inclusion in Liver Cancer Research Databases in the United States

**DOI:** 10.1007/s40615-024-02178-8

**Published:** 2024-09-23

**Authors:** Gabriela Arroyo Figueroa, Tim F. Greten, Cecilia Monge Bonilla

**Affiliations:** Gastrointestinal Malignancies Section, Thoracic and Gastrointestinal Malignancies Branch, Center for Cancer Research, National Cancer Institute (NCI), National Institutes of Health, Building 10 Rm 8D44E, 9000 Rockville Pike, Bethesda, MD 20892 USA

**Keywords:** Hepatocellular carcinoma, Intrahepatic cholangiocarcinoma, Hispanic population, Cancer genomic databases, Health disparities, Social determinants of health

## Abstract

Primary liver cancer, including hepatocellular carcinoma and intrahepatic cholangiocarcinoma was the sixth leading cause of cancer death in the United States in 2023. Hispanic people constitute approximately 19% of the nation’s total population according to the US Census. Hispanic patients have the highest relative incidence rates of liver cancer compared to non-Hispanic Whites and non-Hispanic Blacks, a disparity frequently overlooked in cancer research. In this study, our primary objective was to analyze the potential underrepresentation of Hispanic individuals in liver cancer research databases. We identified databases that had liver cancer-specific studies and be population-based in the United States. Our search yielded 7 cancer genomic databases, which were analyzed according to incidence percentages across ethnicity and race categories. Our study included 3104 patients; ethnic data was not reported for 13.1% (*n* = 406) of the patients. Samples were predominantly from individuals who identified as Not Hispanic (81.0%), Hispanic individuals represented 5.9%. Race was reported as follows: non-Hispanic Whites (61.0%), Asians (22.0%), non-Hispanic Blacks (5.4%), Other (3.1%), Native American/American Indian/Alaska Native (0.4%), Pacific Islander/Native Hawaiian (0.2%) and not reported (7.9%). These findings collectively underscore significant disparities in the representation of ethnic and racial groups, particularly Hispanics. Given the present racial and ethnic demographics of the US population and the projected surge in the Hispanic population in forthcoming years, it becomes imperative to address health disparities that may worsen without efforts to enhance proper inclusion in cancer research.

## Introduction

**Fig. 1:**
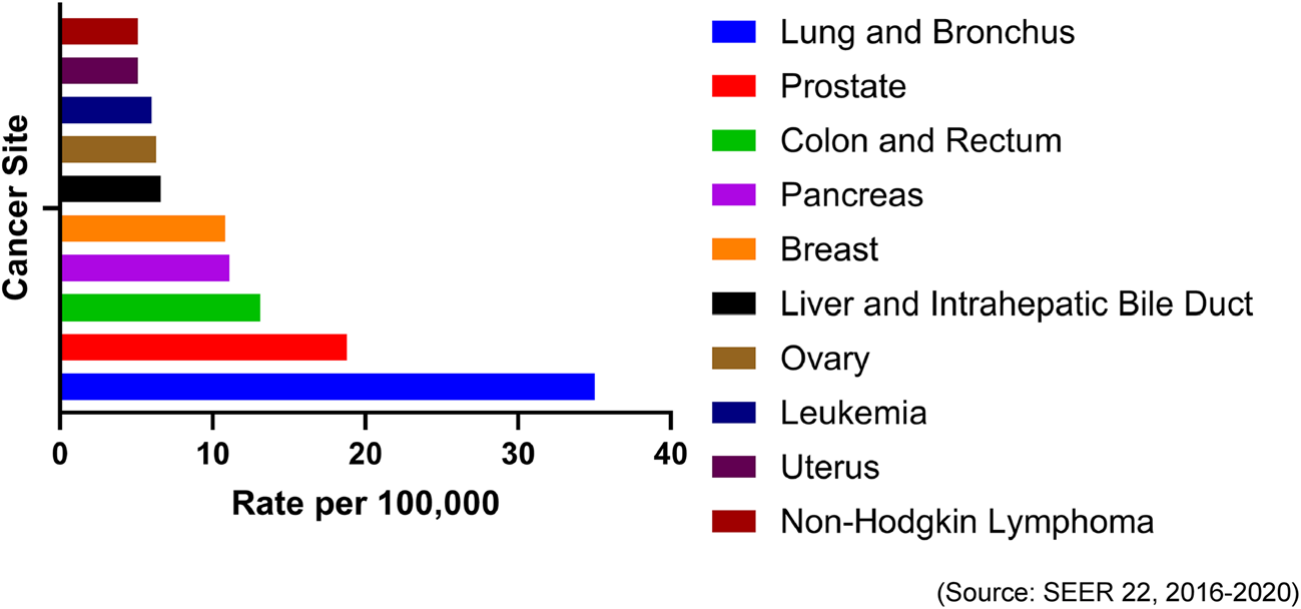
Mortality of the 10 leading cancer sites over a 5-year span in the US population

Primary liver cancer is the term used for cancer originating in the liver. The predominant form of primary liver cancer seen in adults is hepatocellular carcinoma (HCC). Bile duct cancer, known as cholangiocarcinoma, originates in the conducts linking the liver and gallbladder to the small intestine and accounts for 10–15% of all primary liver cancer cases [1]. Cancer emerging in the bile ducts within the liver is termed intrahepatic cholangiocarcinoma (ICC) [2]. Liver and intrahepatic cholangiocarcinoma was the sixth leading cause of cancer death in the United States in 2023 [3] (Fig. 1). Liver and ICC is notably more prevalent in men compared to women, with higher rates observed among Hispanic populations. Based on data from 2016 to 2020, the incidence of new cases of liver and intrahepatic cholangiocarcinoma per 100,000 individuals annually, adjusted for age, stood at 21.7 for Hispanic men and 9.0 for Hispanic women [4].

**Fig. 2:**
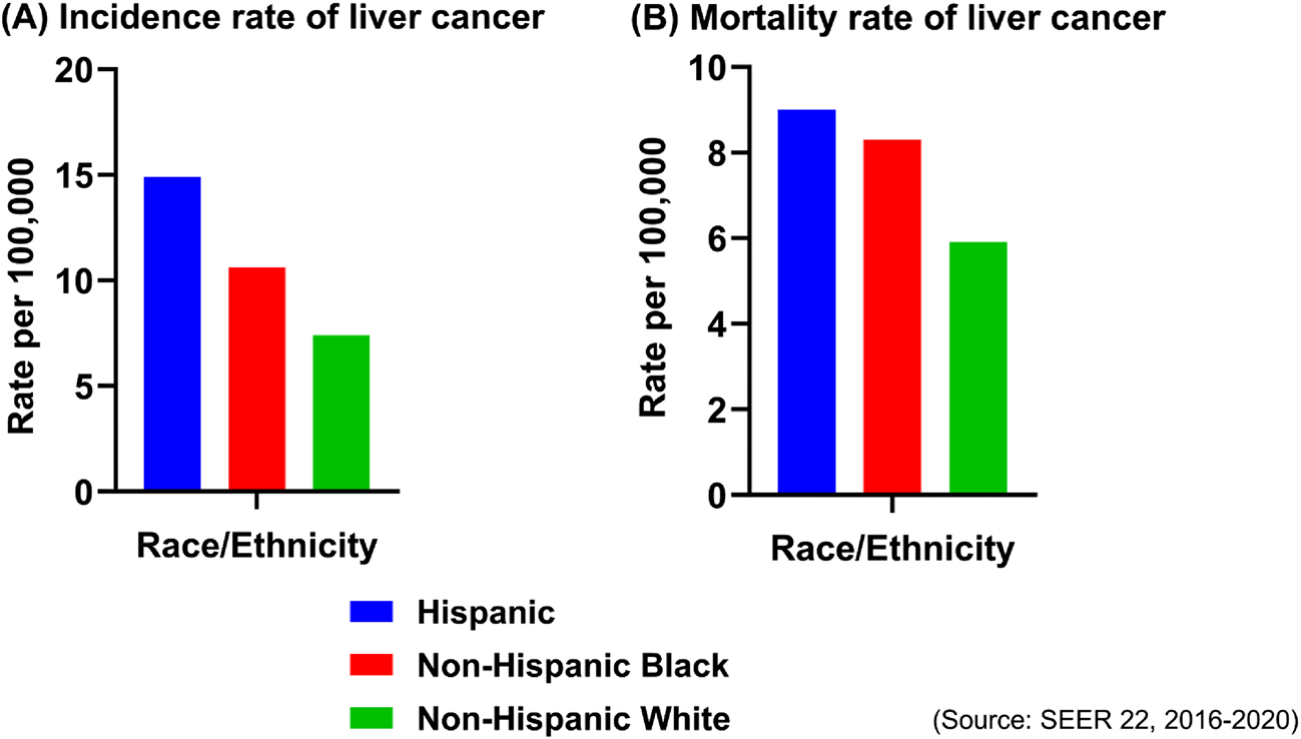
Incidence and mortality rate of liver and intrahepatic cholangiocarcinoma by race and ethnicity

The Hispanic population, defined by the US Office of Management and Budget as a person of Cuban, Mexican, Puerto Rican, South or Central American, or other Spanish culture regardless of their race, constitutes approximately 19.1% (63.7 million) of the nation’s total population according to the 2023 US Census [5, 6]. This demographic group is projected to represent one in every five residents by 2030 [7, 8]. Hispanic patients have the highest relative incidence rate of liver cancer compared to non-Hispanic Whites (NHW) and non-Hispanic Blacks (NHB), a disparity frequently overlooked in cancer research [9, 10] (Fig. 2).

Research indicates a rising incidence of liver cancer among Hispanic men and women, with US-born Hispanic people facing a 45% increased risk of HCC compared to those foreign-born [11]. Among the various ethnic and racial groups affected, Hispanic people face distinct disparities and challenges. Accessing healthcare and treatments poses difficulties for Hispanic individuals who often lack medical insurance coverage and undergo limited preventive medical testing and education. Factors such as language, cultural differences, and economic hardships increase these discrepancies [7]. Projections highlight impending challenges, by 2030, both Hispanic and NHB populations are anticipated to experience the highest incidence rates of HCC, emphasizing the need for tailored interventions and comprehensive healthcare approaches [12].

Various factors contribute to the complex landscape of liver cancer in the United States, affecting diverse demographic groups differently. Research suggests the higher incidence rate of liver cancer among Hispanic individuals compared to NHWs may be linked to experiencing increased rates of risk factors such as: insulin resistance, obesity, type 2 diabetes, heavy alcohol use, smoking, metabolic dysfunction associated fatty liver disease (MAFLD), chronic infections such as Hepatitis B and C, and socioeconomic determinants of health [13–19]. Differences in liver cancer incidence rates between US-born and foreign-born Hispanic individuals highlight the impact of cultural assimilation, with higher rates observed among the US-born due to increased rates of obesity, alcohol consumption, and smoking [19, 20].

Research involving genomic characterization is fundamental in cancer research to advance both diagnostic and treatment approaches tailored to individual tumors. Investigating genomic characteristics and alterations associated with cancer has led to the development of treatment drugs designed to target specific genetic characteristics of malignant cancer cells, minimizing toxicity compared to conventional treatments [21, 22]. Similar to clinical trials ensuring inclusivity across all races and ethnicities, genome cancer research should be inclusive of the population as a whole.

The study by Guerrero et al. (2018) analyzes the representation of different racial and ethnic groups in fundamental and applied cancer research, highlighting potential disparities and underrepresentation that may impact the validity and applicability of research outcomes. Their findings highlighted persistent disparities in the representation of racial and ethnic minorities in cancer clinical trials. Despite efforts such as the National Institutes of Health Revitalization Act of 1993 mandating appropriate representation of minorities in research, the proportion of minorities participating in cancer clinical trials remains lower than their representation in the general population. NHW participants were consistently overrepresented in precision oncology studies for various cancers, while NHB and Hispanic participants were consistently underrepresented [23].

In an analysis of the Phase I and II clinical trials, a database search was conducted in clinicaltrials.gov for interventional liver cancer studies conducted in the United States between September 1, 2002, and February 1, 2023, resulting in the inclusion of 37 trials with 963 total patients. The proportion of patients by race and ethnicity revealed disparities, with higher incidence rates of liver cancer among American Indian/Alaska Native and Hispanic populations. However, less than half of the Phase I or II clinical trials reported race and ethnicity data, indicating significant underrepresentation of NHB and Hispanic populations compared to their relative incidence rates of liver cancer [24].

A subsequent analysis of multinational Phase III advanced liver cancer trials aiming for the approval of The Food and Drug Administration, analyzed the enrollment of Hispanic patients to assess ethnic and racial representation. A database search from July 1, 2012, to July 31, 2022, including PubMed, Embase, and Web of Science identified thirteen studies for analysis. Despite Hispanic individuals having the highest relative incidence rates of liver cancer among major racial and ethnic groups, they were significantly underrepresented in these trials, comprising only 1.6% of patients compared to 31% NHW patients and 47% Asian patients over the past decade [25].

These studies emphasize that understanding and addressing these disparities are essential steps toward promoting diversity, equity, and inclusivity in cancer research. Our aim with this study is to analyze the potential underrepresentation of Hispanic individuals in liver cancer research databases, taking into consideration the representation of all the Hispanic population in all phases of liver cancer clinical trials. We compare the incidence rates of liver cancer across diverse racial and ethnic groups within the most cancer genome databases.

## Methods

### Selection and Databases Used to Analyze Ethnic and Racial Groups

We analyzed incidence rates by ethnicity and race from cancer genomic databases, focusing on liver cancer, particularly HCC and ICC, within the United States. Our decision to focus the study specifically on HCC and ICC, rather than including gallbladder and extrahepatic cholangiocarcinoma stems from several important considerations. Firstly, HCC and ICC are often grouped together in statistical analysis due to their and burden, as seen in NCI’s Surveillance, Epidemiology and End Results (SEER) program. Grouping these cancers in statistical analyses aids in the understanding of liver cancer epidemiology and risk factors.

Databases in our analysis had to contain liver cancer-specific studies and be population-based in the United States. Our search yielded nine databases; however, two were excluded due to their focus on proteogenomic datasets from the National Cancer Institute’s Proteomic Data Commons (NCI PDC) and medical image archives NCI’s The Cancer Imaging Archive (TCIA) [26, 27].

The databases for analysis were: The National Cancer Institute’s Patient-Derived Models Repository (NCI PDMR), Genomics Data Commons (GDC), cBioPortal for Cancer Genomics, American Association for Cancer Research’s [AACR] Project GENIE v.16.0, International Cancer Genome Consortium (ICGC) Data Portal, HCCDB: Integrative Molecular Database of Hepatocellular Carcinoma, and Liver Cancer Expression Resource (CancerLivER) [28–36]. Table 1 provides specific information about the databases, such as primary focus, data types, liver cancer studies, populations from which the databases derive their cases, and accessibility. Within these databases, ethnicity was categorized as Hispanic, Not Hispanic, or Not reported, while race was categorized as NHW, NHB, Asian, Native American (NA), Pacific Islander (PI), Native Hawaiian/Pacific Islander (NH/PI), American Indian/Alaska Native (AI/AN), Other or Not reported. Patients who declined to disclose their race and ethnicity were included in the Not reported category. We analyzed incidence percentages across ethnicity and race categories, comparing these among databases, and examining the representation of Hispanic patients.

**Table 1:** Comparative analysis of cancer genomics databases

Database	Primary focus	Data types	Liver cancer studies	Populations	Access
NCI PDMR	Patient-derived models	PDXs, PDCs, CAFs, PDOrgs	Liver/hepatobiliary cancer studies	Primarily US	Open access
NCI GDC	Genomic data	DNA sequencing, RNA sequencing, methylation, clinical annotations	The Cancer Genome Atlas: Liver Hepatocellular Carcinoma and cholangiocarcinoma	Primarily US	Open access
cBioPortal	Cancer genomic data	Genomic data (mutations, copy number alterations, gene expression, methylation), clinical data	LIHC (TCGA, Firehouse Legacy), LIHC (TCGA, PanCancer Atlas), and HCC (MSK, JCO Precis Oncol 2023)	Primarily US, with some international samples	Open access
AACR Project GENIE v.16.0	Genomic data from cancer patients	Genomic data (DNA, RNA), clinical data	HCC, HCC + ICC, ICC	Global, with the ability to separate US samples	Open access
ICGC Data Portal	Sequencing and genomic data of liver cancer patients	Genomic data (DNA, RNA), clinical data	HCC	Primarily US, with samples from other countries	Open access
HCCDB	Hepatocellular carcinoma	Genomic data (DNA, RNA, methylation), clinical data	HCC-specific	Primarily US	Open access
CancerLivER	Liver cancer gene expression	Gene expression data (mRNA, microRNA)	Liver cancer-specific	Primarily US, but may include samples from other regions	Open access

## Results

We collected ethnic and racial data from six publicly accessible cancer genomic databases; NCI’s GDC Data Portal, cBioPortal for Cancer Genomics, AACR Project GENIE, NCI’s PDMR, ICGC Data Portal, HCCDB: Integrative Molecular Database of Hepatocellular Carcinoma and Liver Cancer Expression Resource (CancerLivER). We found that 66% of these databases provided clinical data regarding the ethnicity and race of patients; NCI’s GDC Data Portal, cBioPortal, AACR Project GENIE and NCI’s PDMR.

The NCI GDC Data Portal includes 17,511 cases: liver and intrahepatic cholangiocarcinoma account for 2.4% (*n* = 420). This data stems from programs including The Cancer Genome Atlas (TCGA) and Human Cancer Models Initiative (HCMI). Ethnic and racial data from 418 cases sourced from the TCGA program is available. The HCMI project does not currently include ethnic or racial data and is excluded from this analysis.

**Fig. 3:**
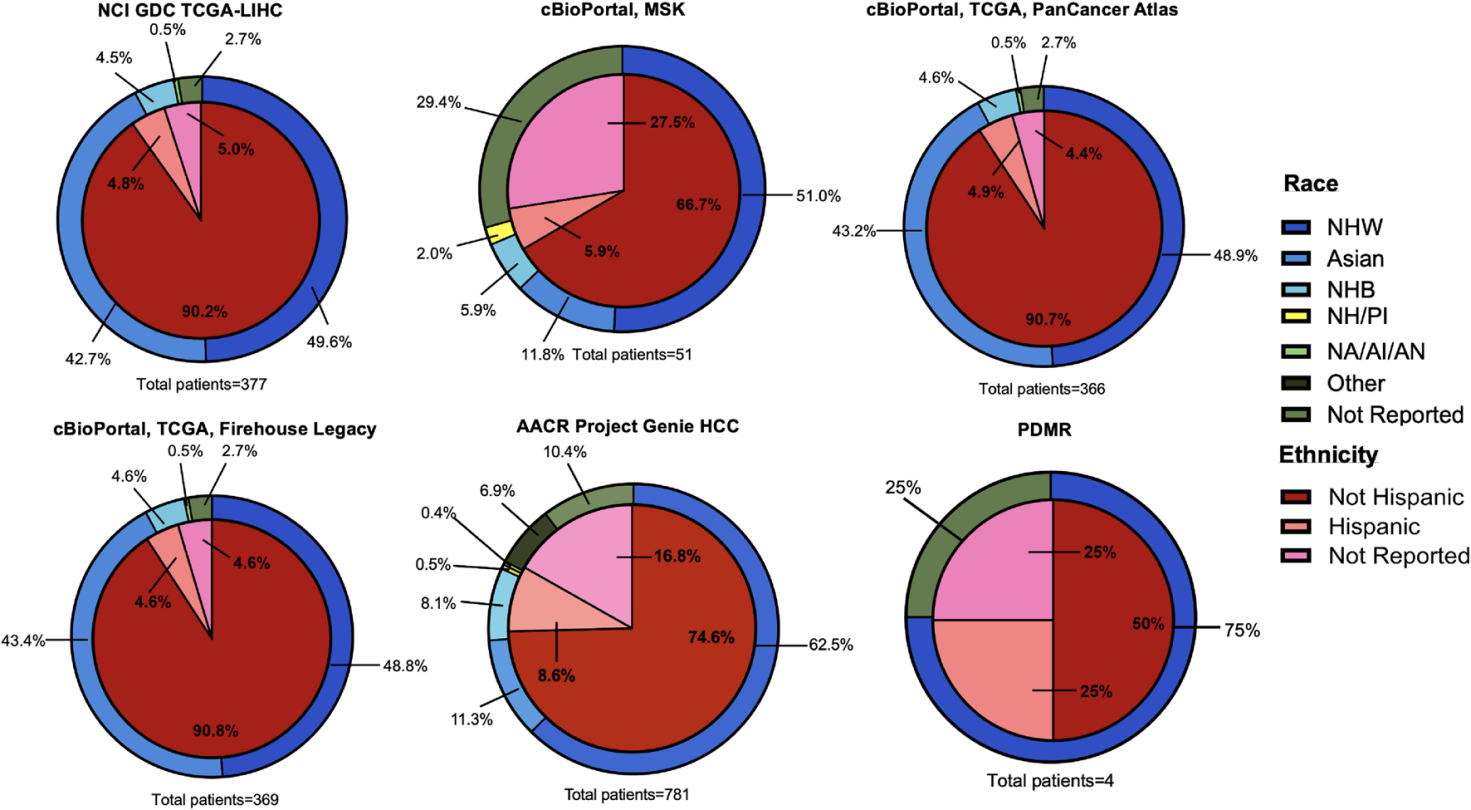
Hepatocellular carcinoma research databases results by race and ethnicity

The TCGA program consists of two branches within the GDC Data Portal: Liver Hepatocellular Carcinoma (LIHC) and cholangiocarcinoma (CHOL), including liver and intrahepatic bile ducts as primary sites. The TCGA-LIHC dataset, consisting of 377 patients; 90.2% identify as not Hispanic, 5.0% did not report ethnicity, and 4.8% identify as Hispanic. Regarding race distribution, 49.6% are NHW, 42.7% are Asian, 4.5% are NHB, 2.7% did not report race, and 0.5% are AI/AN (Table 2) (Fig. 3).

**Table 2:** Ethnic and racial information by Cancer Genomic Database (National Cancer Institute’s Genomic Data Commons, cBio Portal for Cancer Genomics, American Association for Cancer Research’s Project GENIE, and National Cancer Institute’s Patient-Derived Models Repository)

Project/Cancer type	Patients	Ethnicity						
HCC & Liver/hepatobiliary cancer	4	Not Hispanic	Not reported	**Hispanic**				
		2 (50%)	1 (25%)	**1** **(25%)**				
		Race						
		NHW	Asian	Not reported	NHB	Other	PI	NA
		3 (75%)	0	1 (25%)	0	0	0	0
**Database: NCI GDC**								
TCGA-LIHC	377	Not Hispanic	Not reported	**Hispanic**				
		340 (90.2%)	19 (5.0%)	**18** **(4.8%)**				
		Race						
		NHW	Asian	Not reported	NHB	Other	PI	NA
		187 (49.6%)	161 (42.7%)	10 (2.7%)	17 (4.5%)	0	0	2 (0.5%)
TCGA-CHOL	41	Not Hispanic	Not reported	**Hispanic**				
		38 (92.7%)	1 (2.4%)	**2** **(4.9%)**				
		Race						
		NHW	Asian	Not reported	NHB	Other	PI	NA
		35 (85.4%)	3 (7.3%)	0	3 (7.3%)	0	0	0
**Database: cBio Portal for Cancer Genomics**								
Liver Hepatocellular Carcinoma (TCGA, Firehose Legacy)	366	Not Hispanic	Not reported	**Hispanic**				
		332 (90.7%)	16 (4.4%)	**18** **(4.9%)**				
		Race						
		NHW	Asian	Not reported	NHB	Other	PI	NA
		179 (48.9%)	158 (43.2%)	10 (2.7%)	17 (4.6%)	0	0	2 (0.5%)
Liver Hepatocellular Carcinoma (TCGA, PanCancer Atlas)	369	Not Hispanic	Not reported	**Hispanic**				
		335 (90.8%)	17 (4.6%)	**17** **(4.6%)**				
		Race						
		NHW	Asian	Not reported	NHB	Other	PI	NA
		180 (48.8%)	160 (43.4%)	10 (2.7%)	17 (4.6%)	0	0	2 (0.5%)
Hepatocellular Carcinoma (MSK, JCO Precis Oncol 2023)	51	Not Hispanic	Not reported	**Hispanic**				
		34 (66.6%)	14 (27.5%)	**3** **(5.9%)**				
		Race						
		NHW	Asian	Not reported	NHB	Other	PI	NA
		26 (51%)	6 (11.8%)	15 (29.4%)	3 (5.9%)	0	1 (2%)	0
Total	786	Not Hispanic	Not reported	**Hispanic**				
		701 (89.2%)	47 (6%)	**38** **(4.8%)**				
		Race						
		NHW	Asian	Not reported	NHB	Other	PI	NA
		385 (49%)	324 (41.3%)	35 (4.5%)	37 (4.7%)	0	1 (0.1%)	4 (0.5%)
**Database: AACR Project GENIE v.16.0**								
Hepatocellular carcinoma	781	Not Hispanic	Not reported	**Hispanic**				
		583 (74.6%)	131 (16.8%)	**67** **(8.6%)**				
		Race						
		NHW	Asian	Not reported	NHB	Other	PI	NA
		488 (62.5%)	88 (11.3%)	81 (10.4%)	63 (8.1%)	54 (6.9%)	4 (0.5%)	3 (0.4%)
Hepatocellular carcinoma plus Intrahepatic cholangiocarcinoma	60	Not Hispanic	Not reported	**Hispanic**				
		50 (83.3%)	6 (10.0%)	**4** **(6.7%)**				
		Race						
		NHW	Asian	Not reported	NHB	Other	PI	NA
		43 (71.7%)	6 (10.0%)	5 (8.3%)	2 (3.3%)	3 (5.0%)	0	1 (1.7%)
Intrahepatic cholangiocarcinoma	1082	Not Hispanic	Not reported	**Hispanic**				
		911 (84.2%)	101 (9.3%)	**70** **(6.5%)**				
		Race						
		NHW	Asian	Not reported	NHB	Other	PI	NA
		806 (74.5%)	82 (7.6%)	74 (6.8%)	59 (5.5%)	55 (5.1%)	2 (0.2%)	4 (0.4%)
Total	1923	Not Hispanic	Not reported	**Hispanic**				
		1544 (80.3%)	238 (12.4%)	**141** **(7.3%)**				
		Race						
		NHW	Asian	Not reported	NHB	Other	PI	NA
		1337 (69.5%)	176 (9.2%)	160 (8.3%)	124 (6.4%)	112 (5.8%)	6 (0.3%)	8 (0.4%)

**Fig. 4:**
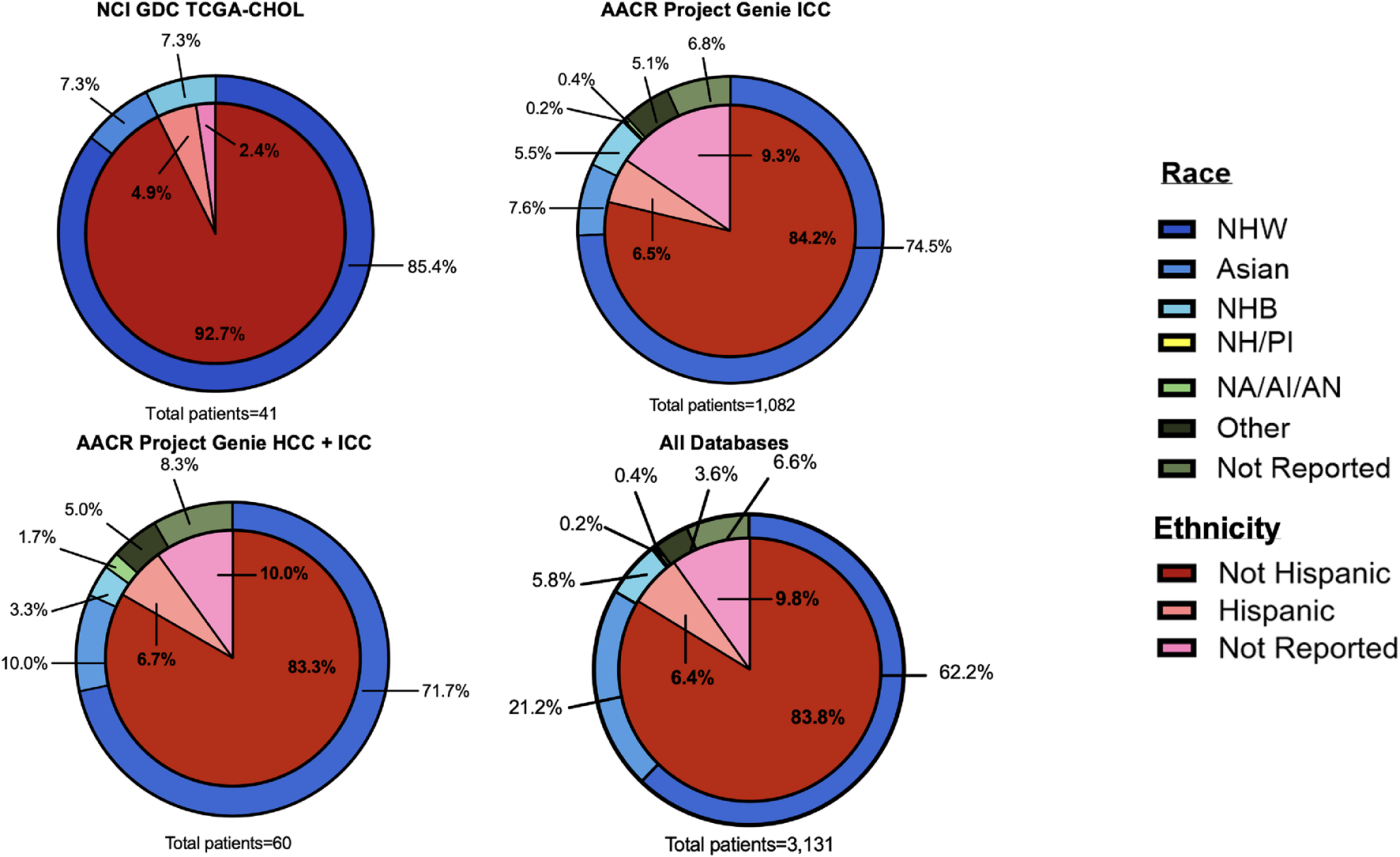
Liver cancer research databases: analysis of hepatocellular carcinoma, intrahepatic cholangiocarcinoma, and combined results by race and ethnicity

Similarly, within the TCGA-CHOL dataset, which includes 41 patients, 92.7% identify as not Hispanic, 4.9% identify as Hispanic, and 2.4% did not report ethnicity. The racial composition consisted of NHW accounting for 85.4%, Asians 7.3%, and NHB for 7.3% of the dataset (Table 2) (Fig. 4).

The cBioPortal registry, includes 11 liver cancer studies, ethnicity and racial data is provided for three studies: LIHC (TCGA, Firehouse Legacy), LIHC (TCGA, PanCancer Atlas), and HCC (MSK, JCO Precis Oncol 2023). Table 2 presents detailed ethnic and racial information for each of the studies (Table 2) (Fig. 3). Ethnic and racial characteristics are reported for 94% (*n* = 739) of the patients within the database. The majority of patients (89.2%), identify as not Hispanic, 6% did not report ethnicity, and 4.8% identify as Hispanic (Table 2). Forty-nine percent of patients identify as NHW, 41.3% as Asian, 4.7% as NHB, 4.5% are not reported, 3.6%and 0.6% identify as NH/PI (Table 2).

Additionally, in the AACR Project GENIE ethnic, and racial data for patients with liver cancer and hepatobiliary cancers was reported for one study: GENIE Cohort v.16.0-public (1,923 patients). The following US participating centers within the database were chosen for analysis; Dana-Farber Cancer Institute, Johns Hopkins Sidney Kimmel Comprehensive Cancer Center, Memorial Sloan Kettering Cancer Center, The University of Texas MD Anderson Cancer Center, Vanderbilt-Ingram Cancer Center, Children’s Hospital of Philadelphia, Duke Cancer Institute-Duke University Health System, The Herbert Irving Comprehensive Cancer Center-Columbia University, Providence Health & Services Cancer Institute, Swedish Cancer Institute, University of California, Wake Forest Baptist Medical Center-Wake Forest University Health Sciences, Yale Cancer Center-Yale University, and University of Chicago Comprehensive Cancer Center.

The following cancer types within primary liver cancer are analyzed for this study, HCC, HCC plus ICC, and ICC. For HCC as the primary cancer type, 781 patients are included.; 74.6% of patients identify as not Hispanic, 16.8% did not report ethnicity, and 8.6% identify as Hispanic. The racial distribution among patients is: 62.5% NHW, 11.3% Asian, 8.1% NHB, 10.4% did not report race, 6.9% are reported as other, 0.5% are PI, and 0.4% are NA (Table 2) (Fig. 3).

Moreover, results for HCC plus ICC include; 83.3% identify as non-Hispanic, 10.0% did not report ethnicity, and 6.7% of patients identify as Hispanic. Race information is reported as: 71.7% NHW, 10.0% Asian, 8.3% did not report race, 3.3% NHB, 5.0% other, and 1.7% NA. Similarly, for ICC, 84.2% of patients identify as non-Hispanic, 9.3% did not report ethnicity, and 6.5% are Hispanic. Racial distribution consists of 74.5% NHW, 7.6% Asian, 6.8% not reported, 5.5% NHB, 5.1% as other, 0.4% as NA, and 0.2% as PI (Table 2) (Fig. 4).

Ethnic and racial characteristics were collected from NCI’s PDMR. This database includes a total of 1197 cancer cell lines and tumor samples. Among these, four samples are associated with liver cancer, 50% (*n* = 2) for HCC and 50% (*n* = 2) for liver/hepatobiliary cancer. Our analysis revealed that 75% of the patients (*n* = 3) had ethnic and racial data; all identify as NHW, and one identified as Hispanic (Table 2) (Fig. 3).

The ICGC Data Portal, HCCDC (Integrative Molecular Database of Hepatocellular Carcinoma), and CancerLivER databases do not include ethnic and racial data, thereby limiting the capacity to assess the representation of Hispanic individuals within them.

Within the databases analyzed, our study included 3131 patients. No ethnic data was reported for 9.8% (*n* = 306) of the patients analyzed. Samples were predominantly from individuals who identified as Not Hispanic (83.8%), Hispanic individuals represented 6.4%. Race is reported as follows: NHWs (62.2%), Asians (21.2%), not reported (6.6%), NHBs (5.8%), other (3.6%), American/American Indian/Alaska Native (0.4%), and Pacific Islander/Native Hawaiian (0.2%) (Table 3) (Fig. 4).

**Table 3:** Ethnic and racial information for all Cancer Genomics Databases (National Cancer Institute’s Genomic Data Commons, cBio Portal for Cancer Genomics, American Association for Cancer Research’s Project GENIE, and National Cancer Institute’s Patient-Derived Models Repository)

Study	Patients	Ethnicity						
Total for all databases	3131	Not Hispanic	Not reported	**Hispanic**				
		2625 (83.8%)	306 (9.8%)	**200** **(6.4%)**				
		Race						
		NHW	Asian	Not reported	NHB	Other	PI	NA
		1947 (62.2%)	664 (21.2%)	206 (6.6%)	181 (5.8%)	112 (3.6%)	7 (0.2%)	14 (0.4%)

## Discussion

The findings from our study analyzing the selected databases collectively underscore significant disparities in the representation of ethnic and racial groups, particularly Hispanics, within basic cancer research such as patient-derived models and cancer genomics. In cancer research, genomic datasets derived from human tumor specimens or experimental models facilitate the validation of findings in clinical contexts [37]. In the field of precision medicine, disparities in cancer care outcomes have been an area of significant research and discussion. These disparities are often attributed to variations in healthcare delivery systems, as well as differences in tumor biology and genetics based on race and ethnicity. There is still a lack of comprehensive evidence on the role of precision medicine in specific cancer types among diverse populations [38, 39]. Factors such as socioeconomic status, geographic location, access to healthcare facilities, and representation in clinical trials can significantly impact an individual’s ability to benefit from precision medicine [40].

Hispanic individuals face significant underrepresentation in liver cancer genome databases, reflecting the existing disparities which may be largely influenced by healthcare access and social determinants, such as, mistrust in the medical system, and socioeconomic inequalities [25]. Hispanic patients from low socioeconomic status neighborhoods, face an increased risk of developing HCC compared to those in higher socioeconomic status areas [41]. This correlation often leads to larger tumor presentations and a higher likelihood of receiving nonsurgical interventions for HCC partially attributable to reduced health insurance coverage [12, 42, 43].

Structural racism, alongside institutional barriers and minority distrust of research may exacerbate disparities experienced by Hispanic patients in cancer precision medicine initiatives. This systemic issue encompasses explicit and implicit rules, laws, and institutional norms that perpetuate inequitable social structures [44–46]. Additionally, factors such as distrust of the medical system, lack of information, and limited knowledge about disease progression and treatment options further hinder the participation of Hispanic individuals in cancer research [47–52].

Studies using SEER Medicare data have revealed that NHB and Hispanic patients are significantly less likely than NHW patients to receive HCC screening tests and less likely to be referred to specialty clinics, often receiving diagnoses at later stages, leading to fewer treatment options and a poorer prognosis in comparison to NHW individuals [12, 53]. Hispanic and NHB patients with advanced HCC, along with are less likely to receive treatment with immunotherapy and palliative care, along with a significant proportion of racial and ethnic minority Medicare beneficiaries foregoing hospice care in their final year of life [54, 55].

In contrast, the overrepresentation of NHWs in liver cancer research may reflect systemic privileges, and structural advantages within the healthcare and research systems. NHWs often have greater access to healthcare resources, socioeconomic opportunities, and education compared to minority populations, enabling them to participate more readily in research studies. Moreover, systemic biases and institutional practices that prioritize the needs and experiences of NHW individuals may inadvertently exclude or marginalize minority communities from research opportunities [56, 57].

Notably, not all states submit Hispanic data to the SEER program, particularly those with substantial Hispanic populations. This lack of representation is augmented by the diverse ethnic backgrounds within the Hispanic community, which are often overlooked in genomic studies [58]. Previous studies have indicated that the SEER program’s coverage does not adequately reflect South American and Caribbean populations, which together comprise a significant percentage of the Hispanic demographic in the United States [59]. Consequently, the data derived from SEER may not accurately represent the incidence and outcomes of liver cancer in these populations.

Research indicates that Hispanic patients are more likely to be diagnosed at advanced stages of liver cancer, which correlates with worse survival rates [60]. While some studies report a decrease in HCC incidence, this trend may not be uniformly applicable across all demographics. For instance, while there was a reported decline in HCC incidence among younger age groups, older groups, those aged 50 and above, have shown increasing rates of liver cancer [61]. Additionally, the increasing prevalence of risk factors such as Hepatitis C, which is notably high among certain Hispanic subpopulations, can continue to contribute to rising liver cancer rates [62].

The underrepresentation of Hispanic populations in cancer registries like SEER, combined with challenges related to data quality and the complexities of ethnic diversity, complicates the understanding of liver cancer trends. Future research must address these disparities by improving data collection methods and ensuring that diverse Hispanic populations are adequately represented in cancer research and clinical trials [25, 63].

Efforts to address liver cancer among Hispanic individuals require multifaceted strategies focusing on prevention, early detection, treatment, and improved access to healthcare services [64]. Addressing these disparities requires enhanced recruitment strategies, and policy interventions to promote diversity and inclusivity. Implementing processes which engage research populations to understand their concerns regarding research agendas, can play an important role in fostering trust within minority communities who may face discrimination [47, 48]. Changes in mandatory minimal requirements within clinical research such as the detailed reporting of racial and ethnic characteristics, and recruitment can improve the ability to address health disparities. Additionally, it is not known how race and ethnicity is assessed or captured, such as by self-report or determined by a researcher, this is something that should be standardized across institutions to maintain uniformity [47].

Utilizing genetic ancestry for the collection of race and ethnicity data has demonstrated to be a more accurate and likely improved method compared to self-reported race and ethnicity in understanding patient’s genetic backgrounds [65]. Within the Hispanic population, individuals often exhibit diverse racial ancestries. By discerning the predominant ancestral components within specific patients, researchers can better understand varying genetic backgrounds and their implications in oncologic diseases [66–68].

However, it is crucial to recognize that genetic information, while invaluable in cancer studies, should not be combined with race and ethnicity, especially in the context of disparities research. Both genetic ancestry, race, and ethnicity are valuable and necessary, yet they measure different aspects of human experience and risk. The All of Us Research Program emphasizes the importance of using genetic ancestry as a distinct variable, advocating against the use of race as a proxy for genetic ancestry due to the potential for misrepresentation and oversimplification [69–71].

The combination of these concepts can lead to discrimination and prolong existing health disparities, particularly among minority populations. Therefore, researchers must approach the integration of genetic ancestry and race and ethnicity data with caution, ensuring that each is considered in its appropriate context. While it provides a more accurate way to view genetic backgrounds, it is imperative to maintain a clear distinction between genetic data and social constructs of race and ethnicity. This consideration is essential to avoid reinforcing stereotypes and to promote equitable healthcare practices [72]. By employing a detailed understanding of genetic ancestry, researchers can better address the complexities of health disparities and improve outcomes for diverse populations [73].

Cancer research based on ethnicities is crucial to addressing health disparities effectively [74, 75]. Understanding the distinct health burdens within specific populations is imperative for pinpointing underlying causes. Insufficient data hampers the accurate understanding of liver cancer and limits the development of tailored treatment approaches. By acknowledging and actively working to mitigate these disparities, the healthcare community can strive toward a more inclusive and patient-centered approach to precision medicine. This proactive stance is essential for improving outcomes for all cancer patients, irrespective of their background or circumstances, to ensure that research breakthroughs and personalized treatments benefit all individuals equitably.

This study describes a notable pattern that emerges across cancer genome databases, revealing a consistent underrepresentation of Hispanic populations in the context of liver cancer in genomic databases. We emphasize the urgent need to enhance diversity in genomic studies, ensuring a more accurate representation of the population, and promoting equitable advancements in liver cancer research**.**

The limitations of this study include factors such as, reliance on secondary data sources, which may introduce biases or inaccuracies in the original data collection methods, which may not accurately reflect the true demographic composition of the Hispanic population. This limitation restricts the depth of our understanding regarding the outcomes of Hispanic individuals with liver cancer within these datasets.

With our study’s findings regarding the underrepresentation of Hispanic individuals in liver cancer research databases, it is evident that disparities in healthcare access, socioeconomic factors, and systemic inequities significantly contribute to this issue [24]. To address these disparities and improve minority representation, particularly among the Hispanic population, targeted interventions should focus on reforming patient recruitment strategies and making mandatory the detailed reporting of race and ethnicity for all patients to ensure diversity and accurate population representation within research [76–78]. By understanding the complexities surrounding Hispanic representation in liver cancer research studies, we can develop tailored interventions aimed at enhancing access to healthcare, fostering trust in the medical system, and engaging diverse communities in research. Greater transparency and accessibility of ethnicity data in research databases are essential for addressing health disparities, tailoring interventions to meet the needs of diverse patient populations, and the overall improvement of cancer research.

In conclusion, addressing disparities in precision oncology diagnostics and treatment is paramount for achieving health equity in cancer care. These disparities highlight critical issues regarding inclusivity, diversity, and equity cancer research efforts, emphasizing the imperative for more comprehensive and representative datasets. By acknowledging and actively working to mitigate these disparities, healthcare providers and researchers can pave the way for a more inclusive and effective approach to precision oncology. Collaborative efforts to increase diversity in research studies and enhance access to precision oncology for all individuals are essential steps towards reducing disparities and improving outcomes in cancer care.
